# Influence of Oxidative Stress on Stored Platelets

**DOI:** 10.1155/2016/4091461

**Published:** 2016-02-02

**Authors:** K. Manasa, R. Vani

**Affiliations:** Department of Biotechnology, Center for Post Graduate Studies, Jain University, No. 18/3, 9th Main, 3rd Block, Jayanagar, Bangalore 560011, India

## Abstract

Platelet storage and its availability for transfusion are limited to 5-6 days. Oxidative stress (OS) is one of the causes for reduced efficacy and shelf-life of platelets. The studies on platelet storage have focused on improving the storage conditions by altering platelet storage solutions, temperature, and materials. Nevertheless, the role of OS on platelet survival during storage is still unclear. Hence, this study was conducted to investigate the influence of storage on platelets. Platelets were stored for 12 days at 22°C. OS markers such as aggregation, superoxides, reactive oxygen species, glucose, pH, lipid peroxidation, protein oxidation, and antioxidant enzymes were assessed. OS increased during storage as indicated by increments in aggregation, superoxides, pH, conjugate dienes, and superoxide dismutase and decrements in glucose and catalase. Thus, platelets could endure OS till 6 days during storage, due to the antioxidant defense system. An evident increase in OS was observed from day 8 of storage, which can diminish the platelet efficacy. The present study provides an insight into the gradual changes occurring during platelet storage. This lays the foundation towards new possibilities of employing various antioxidants as additives in storage solutions.

## 1. Introduction

Platelets play an important role in maintaining hemostasis. Platelet transfusions are essential for treatment of patients with thrombocytopenia and are routinely used during surgery, chemotherapy, and treatment of various bleeding disorders. However, platelet storage* in vitro* and their availability for transfusion are limited to 5-6 days at 22°C, during which platelets lose their viability and activity [1]. Changes occur in morphology, adhesion and aggregation, membrane features, and the activation and apoptotic markers during prolonged storage [1, 2].

Banked platelets begin to lose their function, which can be due to (i) the platelet activation during preparation and storage process or (ii) the changes in pH and enzyme activation of the plasma environment or (iii) both phenomena [3]. Oxidative stress (OS) is also one of the causative factors for reduced efficacy and shelf-life of stored platelets. OS leads to decrease in platelet nitric oxide which increases platelet activation and cellular production of reactive oxygen species (ROS) [4]. But whether resulting oxidative changes trigger activation and apoptosis or activation and apoptosis trigger oxidative changes during storage is still unclear [5].

There are studies on improving the storage conditions by altering platelet storage solutions, storage materials (UPX80 [6], tri-ethylhexyl-trimellitate [7], polyolefin [8], and ELX container [9], etc.), and temperatures 4–37°C [10–21]. Few studies have reported that glucose can be a good additive for new storage formulations [22–24]. Platelet additive solutions (PAS) with different formulations have been tested and seem to be desirable over plasma for the storage of platelet concentrates [10, 25–32]. Storing platelets in additive solutions (Tyrode's buffer) diminishes the risk of infections or allergic reactions caused by platelet storage in plasma [33].

Nevertheless, platelet survival in different storage solutions is still unclear. Thus, the present study was conducted to investigate the influence of storage on platelets in Tyrode's buffer through OS markers: (i) platelet integrity: platelet aggregation, glucose, and pH; (ii) oxidative stress: superoxides, lipid peroxidation (conjugate dienes and thiobarbituric acid reactive substances (TBARS)), protein oxidation (protein carbonyls and sulfhydryls), and antioxidant enzymes (superoxide dismutase (SOD) and catalase (CAT)).

## 2. Experimental Procedures

Animal care and maintenance were in accordance with the ethical committee regulations.

### 2.1. Chemicals

Cytochrome C (Cyt C) was purchased from Sigma-Aldrich Chemicals (St. Louis, MO, USA). All other chemicals were of reagent grade and organic solvents of spectral grade.

### 2.2. Blood Sampling

According to Rajashekharaiah et al., [34] 4-month-old male* Wistar* rats were used for sampling of blood. Blood was aspirated carefully from the heart into tubes with CPDA-1 (Citrate, phosphate, dextrose, and adenine) solution.

### 2.3. Experimental Design

Platelets isolated from blood of five different rats (*n* = 5) were suspended in Tyrode's buffer (NaCl-140 mM; KCl-2.7 mM; NaH_2_PO_4_-0.4 mM; NaHCO_3_-11.9 mM; D-glucose-11.1 mM) and stored in polypropylene tubes at 22°C for a period of 12 days, without continuous agitation. Estimation of OS was performed by assessing the biomarkers on alternate days of storage, that is, 0, 2, 4, 6, 8, 10, and 12.

### 2.4. Isolation of Platelets

Blood was centrifuged at 1500 rpm for 15 min at room temperature to obtain platelet-rich-plasma (PRP). The PRP was then centrifuged at 4000 rpm for 15 min at 22°C. The resulting platelet pellet was gently resuspended in Tyrode's buffer, pH 7.4 [35].

### 2.5. Platelet Aggregation

Platelet aggregation was measured according to modified method of Born and Cross [36]. Hundred microliters of platelet sample was added to each well of the microtiter plate and incubated at 37°C. The absorbance was recorded at 405 nm in shaking mode for 1.5–2 min. The absorbance is inversely proportional to the aggregation in platelets.

### 2.6. Glucose

The level of glucose in platelets during storage was assessed enzymatically by GOD-POD method as described in the Autospan Gold kit and the absorbance was taken at 546 nm [37].

### 2.7. pH

pH of the samples were checked using Fisher Scientific pH strips [22].

### 2.8. Superoxides

Superoxides in the platelets were measured according to Olas and Wachowicz [38]. Two hundred microliters of Cyt C (160 *μ*M) was added to the platelets and incubated at 37°C. The samples were centrifuged at 3500 rpm for 5 min. Reduction of Cyt C was measured spectrophotometrically at 550 nm. To calculate the molar concentration of superoxide, extinction coefficient for Cyt C of 18,700 M^−1^ cm^−1^ was used.

### 2.9. Conjugate Dienes

Conjugate dienes were measured according to Olas and Wachowicz [38]. Platelet samples were transferred to ether/ethanol (1 : 3 v/v) mixture and vortexed. The mixture was centrifuged at 4000 rpm. The level of conjugate dienes was measured spectrophotometrically at 235 nm.

### 2.10. Thiobarbituric Acid Reactive Substances (TBARS)

TBARS were measured according to Olas et al. [39]. The platelet samples were cooled in an ice bath for 10 min. The samples were then transferred to an equal volume of 20% (v/v) cold trichloroacetic acid (TCA) in 0.6 M HCl and centrifuged at 2000 rpm for 15 min. The supernatant was mixed with 0.2 mL of 0.12 M thiobarbituric acid (TBA) in 0.26 M Tris at pH 7.0. The mixture was incubated in a boiling water bath for 15 min and the absorbance was read at 532 nm.

### 2.11. Protein Carbonyls

Protein carbonyls were measured according to Reznick and Packer [40]. Platelet samples were mixed with 2 mL of 10 mM dinitrophenyl hydrazine (DNPH) in 2.5 M HCl and incubated for 1 h at room temperature. After incubation, 2.5 mL of 20% TCA was added and left in ice for 10 min. The mixture was centrifuged at 3000 rpm and the supernatant was discarded. The protein pellets were washed three times with ethanol : ethyl acetate (1 : 1 v/v). The final precipitate was dissolved in 1 mL of 6 M guanidine HCl in 133 mM Tris. The absorbance was read at 370 nm. The carbonyl content was calculated using absorption coefficient of 22,000 M^−1^ cm^−1^.

### 2.12. Protein Sulfhydryls

The protein sulfhydryls were measured according to Habeeb [41]. Platelet samples were mixed with 1.5 mL of 0.08 M buffer (Na-PO_4_, 0.5 mg mL^−1^ of Na_2_-EDTA and 2% SDS, pH 8.0) and vortexed. Then 0.1 mL of 5, 5′-dithiobis-(2-nitrobenzoic acid) (DTNB) was added. This was incubated at room temperature for 15 min. The absorbance was read at 412 nm. Molar absorptivity of 13,600 M^−1^ cm^−1^ was used to calculate protein sulfhydryls.

### 2.13. Superoxide Dismutase (SOD, EC 1.15.1.1)

SOD in the platelet sample was measured according to Misra and Fridovich [42]. Platelet samples were added to 880 *μ*L of carbonate buffer (0.05 M, pH 10.2, 0.1 mM EDTA). Forty microliters of epinephrine was added to the mixture and the absorbance was measured at 480 nm for 4 min. SOD activity was expressed as the amount of enzyme that inhibits oxidation of epinephrine by 50% which is equal to 1 unit.

### 2.14. Catalase (CAT, EC 1.11.1.6)

Catalase was measured according to Aebi [43]. Ten microliters of absolute ethanol was added to 100 *μ*L of platelet sample and incubated in ice bath for 30 min. After incubation, 240 *μ*L of phosphate buffer was added to the above sample. Two hundred and fifty microliters of 0.066 M H_2_O_2_ was added just before reading and the absorbance was measured at 240 nm. The molar extinction coefficient of 43.6 M^−1^ cm^−1^ was used to determine the catalase activity.

### 2.15. Protein

The protein concentration in the samples was determined according to Lowry et al. [44].

### 2.16. Statistical Analyses

Results are represented as mean ± SE. One-way ANOVA was performed between the groups for all the parameters and the results are considered significant at *P* < 0.05. Tukey-Kramer multiple comparison test was performed using GraphPad Prism 6 Software.

## 3. Results

### 3.1. Platelet Aggregation Increased during Storage

**Figure 1:**
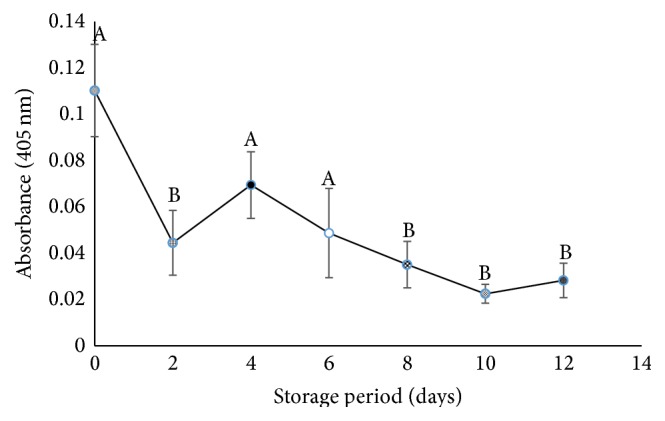
Aggregation in stored platelets. Values are mean ± SE of five animals/group. One-way ANOVA was performed between the groups followed by Tukey-Kramer multiple comparison test, using Graph Pad Prism 6 software, and represented in upper case at *P* < 0.05. Those not sharing the same letters are significantly different.

Aggregation increased significantly by 59% (day 2), 68% (day 8), 79% (day 10), and 74% (day 12) when compared with day 0 at *P* < 0.05 (Figure 1).

### 3.2. Superoxides Increased during Storage

**Figure 2:**
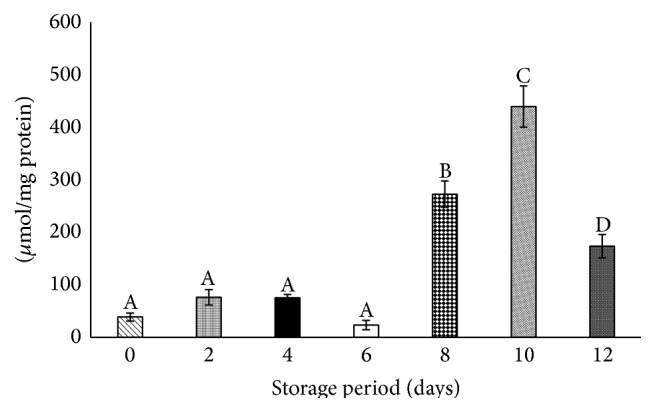
Superoxides in stored platelets. Values are mean ± SE of five animals/group. One-way ANOVA was performed between the groups followed by Tukey-Kramer multiple comparison test, using Graph Pad Prism 6 software, and represented in upper case at *P* < 0.05. Those not sharing the same letters are significantly different.

Superoxides elevated significantly after day 6 of storage. There was an increase of 6-, 10-, and 3-fold on days 8, 10, and 12, respectively, against day 0 at *P* < 0.05. Decrement of 60% was observed on day 12 when compared with day 10 (Figure 2).

### 3.3. Glucose Decreased during Storage

Glucose levels reduced significantly (*P* < 0.05) throughout storage. Glucose levels decreased on day 2 by 39% and were maintained up to day 10. Decline in glucose levels by 91% was observed on day 12 against day 0 (Table 1).

### 3.4. pH Elevated with Storage

pH gradually elevated up to day 10 and by 42% on day 12 against day 0 (*P* < 0.05) (Table 1).

### 3.5. Conjugate Dienes Increased towards End of Storage

Significant increments were observed in conjugate dienes by 24- and 13-fold on days 10 and 12, respectively, with day 0 at *P* < 0.05 (Table 1).

### 3.6. TBARS Increased on Day 10

TBARS levels were maintained till day 8 but incremented by 3-fold on day 10 against day 0 (*P* < 0.05) (Table 1).

### 3.7. Protein Carbonyls Varied during Storage

Carbonyls increased by 9-fold on day 4 against day 0 at *P* < 0.05. Protein carbonyls declined on day 6 against day 4 and were maintained till day 10. Elevation of 6-fold was noted on day 12 against day 0 at *P* < 0.05 (Table 1).

### 3.8. Protein Sulfhydryls Increased after 8 Days of Storage

Protein sulfhydryls augmented only after day 6 and elevation of 1-fold was observed on day 12 against day 0 (*P* < 0.05) (Table 1).

### 3.9. Superoxide Dismutase Incremented after Day 6 of Storage

**Figure 3:**
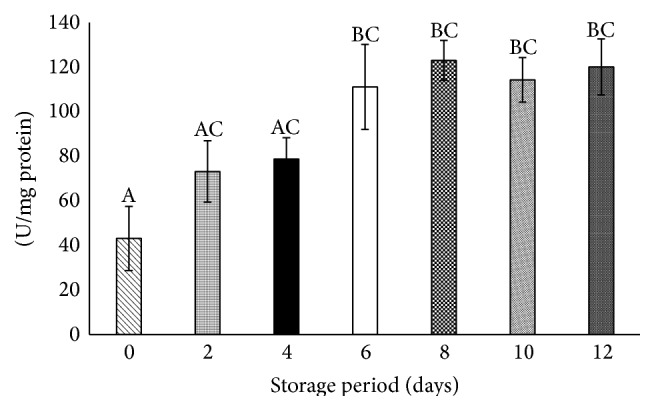
Superoxide dismutase in stored platelets. Values are mean ± SE of five animals/group. One-way ANOVA was performed between the groups followed by Tukey-Kramer multiple comparison test, using Graph Pad Prism 6 software, and represented in upper case at *P* < 0.05. Those not sharing the same letters are significantly different.

SOD significantly increased (*P* < 0.05) by 1-fold on days 6, 8, 10, and 12 with respect to day 0 (Figure 3).

### 3.10. Catalase Declined after 8 Days of Storage

**Figure 4:**
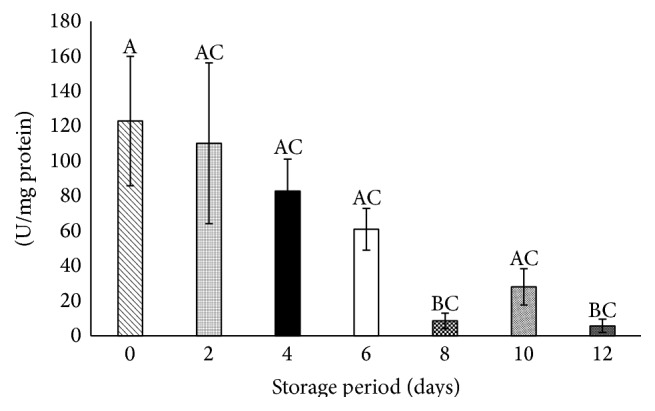
Catalase in stored platelets. Values are mean ± SE of five animals/group. One-way ANOVA was performed between the groups followed by Tukey-Kramer multiple comparison test, using Graph Pad Prism 6 software, and represented in upper case at *P* < 0.05. Those not sharing the same letters are significantly different.

Significant decreases of 93% and 95% were observed on days 8 and 12, respectively, when compared with day 0 at *P* < 0.05 (Figure 4).

## 4. Discussion

Our results showed that platelets could endure OS up to 6 days of storage and the onset of oxidative damage was noted from day 8. Platelets stored in plasma lose their functions considerably and also are exposed to infectious and allergic agents and proteolytic enzymes that can lead to premature clearance from circulation. Storing platelets in Tyrode's buffer provides optimal conditions for cell preservation and helps to control the environmental conditions during storage. Using a defined medium enhances the reproducible quality of stored platelet preparations [33]. The absence of Ca^2+^ in Tyrode's buffer also reduces platelet activation [45]. Platelet aggregation in our study increased in proportion to the free radical generation during storage, as observed in our results of increased superoxides during storage. The decrease in superoxides on day 12 against day 10 can be due to the decrease in number of platelets. The decrease in glucose level is directly proportional to the energy levels and platelet count [24]. The ideal pH for platelet transfusion should be between 6.4 and 7.4 at 22°C [46]. pH increased up to 10.0 by day 12 of storage. This can be due to the depletion of glucose in the medium, as glycolysis produces lactate, which reduces the pH of stored platelets [46]. The factors such as bacterial contamination, storage bag gas permeability [47], and platelet content in storage bag [48] can also affect the pH of platelets during storage.

The primary (conjugate dienes) and secondary (TBARS) products of lipid peroxidation increased at the end of storage. This can be due to elevations in superoxides and other ROS generation beyond the antioxidant defense, as poly-unsaturated fatty acids (PUFAs) are the most oxygen sensitive constituents of cells and easily suffer oxidation [49].

Reactive carbonyl groups in proteins are formed due to oxidation of arginine, lysine, threonine, or proline by the ROS in platelets [50]. The increase in carbonyls on day 4 may be due to the generation of free radicals which in turn triggers the antioxidant defenses (hormesis effect). This was elucidated in our results of day 6 where carbonyls decremented, which may be due to the activation of antioxidant defenses [51]. The elevated carbonyls on day 12 can be due to increased free radicals, overwhelming the platelet antioxidant system.

Sulfhydryls are the potential sites of reversible oxidative modification by S-glutathiolation and S-nitrosylation [52]. The augmented levels of sulfhydryls by the end of storage can be due to the activated or aggregated platelets that are known to exhibit increased surface sulfhydryls, especially protein disulfide isomerase (PDI) sulfhydryls [53]. This is in correlation with our results of increased platelet aggregation during storage.

An increase in SOD activity is the result of elevated free radicals [54]. SOD incremented by day 6 of storage and was maintained till day 12. This is in accordance with the elevation in superoxide levels as observed in our results. The level of superoxide regulates the rate of SOD activity [55]. CAT is activated at higher concentrations of H_2_O_2_ [56] and at lower concentrations, H_2_O_2_ is scavenged by glutathione peroxidase (GSH-Px) [57]. CAT activity decreased by the end of storage though there was an increase in SOD activity which can be due to the H_2_O_2_ scavenging action of GSH-Px.

## 5. Conclusion

Platelets could endure OS till 6 days during storage, due to the antioxidant defense system. An evident increase in OS was observed from day 8 of storage, which can diminish the platelet efficacy.

The present study provides an insight into the gradual changes occurring during platelet storage. This lays the foundation towards new possibilities of employing various antioxidants as additives in storage solutions to attenuate OS and hence enhance the stability and efficacy of stored platelets.

## Figures and Tables

**Table 1:** Glucose, pH, lipid peroxidation, and protein oxidation in stored platelets.

Storage Period (Days)	Glucose (mmol/L)	pH	Lipid peroxidation		Protein oxidation	
			Conjugate dienes (*µ*mol/mg protein)	TBARS (*µ*mol/mg Protein)	Protein carbonyls (*µ*mol/mg protein)	Protein sulfhydryls (*µ*mol/mg protein)
0	12.22 ± 0.7^a^	7.0 ± 0.0^a^	23.20 ± 4.7^a^	9.40 ± 3.1^a^	18.45 ± 5.7^a^	251.45 ± 48.0^ab^
2	7.42 ± 0.6^b^	7.0 ± 0.0^a^	28.26 ± 11.8^a^	11.31 ± 4.8^a^	93.73 ± 25.0^ac^	105.33 ± 30.2^a^
4	5.80 ± 0.1^b^	8.0 ± 0.0^b^	69.64 ± 21.5^a^	4.15 ± 2.3^a^	185.5 ± 21.0^b^	132.26 ± 15.8^a^
6	5.84 ± 0.3^b^	8.4 ± 0.2^b^	21.52 ± 6.6^a^	14.32 ± 5.4^ab^	17.61 ± 5.5^a^	244.57 ± 63.8^ab^
8	7.33 ± 0.6^b^	8.4 ± 0.2^b^	96.6 ± 18.4^a^	7.34 ± 1.4^a^	68.37 ± 3.6^ac^	392.6 ± 46.2^b^
10	6.16 ± 0.1^b^	9.0 ± 0.0^c^	585.7 ± 41.9^b^	38.4 ± 13.0^b^	64.77 ± 14.7^ac^	350.18 ± 37.5^b^
12	1.05 ± 0.2^c^	10.0 ± 0.0^d^	330.9 ± 71.6^c^	4.22 ± 1.0^a^	146.78 ± 33.8^bc^	528.18 ± 55.5^c^

Values are expressed as mean ± SE of five animals/group. Changes between groups were analyzed by one-way ANOVA followed by Tukey-Kramer multiple comparison test, using GraphPad Prism 6 software. *P* < 0.05 was considered significant. Changes between the groups are represented in lower case. Those not sharing the same letters are significantly different.
